# Labour-market marginalisation after mental disorders among young natives and immigrants living in Sweden

**DOI:** 10.1186/s12889-017-4504-4

**Published:** 2017-06-23

**Authors:** Magnus Helgesson, Petter Tinghög, Thomas Niederkrotenthaler, Fredrik Saboonchi, Ellenor Mittendorfer-Rutz

**Affiliations:** 1 Division of Insurance Medicine, Department of Clinical Neuroscience, Karolinska Institutet, SE-171 77, Stockholm, Sweden; 2 Red cross University, College, Stockholm, Box, 1059 141 21 Huddinge, Sweden; 3 Department of Social and Preventive Medicine, Medical University Vienna, Center for Public Health, Spitalgasse 23, 1090 Vienna, Austria

**Keywords:** Mental disorders, Disability pension, Sick leave, Unemployment, Immigrants, Labour-market marginalisation

## Abstract

**Background:**

The aim was to investigate the associations between mental disorders and three different measures of labour-market marginalisation, and differences between native Swedes and immigrants.

**Methods:**

The study comprised 1,753,544 individuals, aged 20–35 years, and resident in Sweden 2004. They were followed 2005–2011 with regard to disability pension, sickness absence (≥90 days) and unemployment (≥180 days). Immigrants were born in Western countries (Nordic countries, EU, Europe outside EU or North-America/Oceania), or in non-Western countries (Africa, Asia or South-America). Mental disorders were grouped into seven subgroups based on a record of in- or specialised outpatient health care 2001–2004. Hazard ratios (HRs) with 95% confidence intervals (CIs) were computed by Cox regression models with both fixed and time-dependent covariates and competing risks. We also performed stratified analyses with regard to labour-market attachment.

**Results:**

Individuals with mental disorders had a seven times higher risk of disability pension, a two times higher risk of sickness absence, and a 20% higher risk of unemployment than individuals without mental disorders. Individuals with personality disorders and schizophrenia/non-affective psychoses had highest risk estimates for having disability pension and long-term sickness absence, while the risk estimates of long-term unemployment were similar among all subgroups of mental disorders. Among persons with mental disorders, native Swedes had higher risk estimates for disability pension (HR:6.6; 95%CI:6.4–6.8) than Western immigrants (4.8; 4.4–5.2) and non-Western immigrants (4.8; 4.4–5.1), slightly higher risk estimates for sickness absence (2.1;2.1–2.2) than Western (1.9;1.8–2.1), and non-Western (1.9;1.7–2.0) immigrants but lower risk estimates for unemployment (1.4;1.3–1.4) than Western (1.8;1.7–1.9) and non-Western immigrants (2.0;1.9–2.1). There were similar risk estimates among sub-regions within both Western and non-Western countries. Stratification by labour-market attachment showed that the risk estimates for immigrants were lower the more distant individuals were from gainful employment.

**Conclusions:**

Mental disorders were associated with all three measures of labour-market marginalisation, strongest with subsequent disability pension. Native Swedes had higher risk estimates for both disability pension and sickness absence, but lower risk estimates for unemployment than immigrants. Previous labour-market attachment explained a great part of the association between immigrant status and subsequent labour-market marginalisation.

## Background

Mental disorders are due to the usual early age of onset, the strong impact on occupational functioning, the relative frequent and increasing occurrence, and the high prevalence of relapses one of the main causes of marginalisation on the labour market [1–3]. Labour market marginalisation, i.e. severe problems in finding and keeping a job, is a growing and serious public health and economic problem among young adults in Europe [4]. In Sweden, the unemployment rate has during the last 10 years been over 20% among young adults 16–24 years of age, which is higher than the average unemployment rate in the European Union [5]. Moreover, the sickness absence rate has historically been high in Sweden, in 2015 around 4% of the Swedish population was estimated to be on sickness absence at any given time, which was more than twice as many as in Denmark, Finland or Iceland [6]. There has also been an increase in the incidence of disability pension among young adults during the last decades: 8700 individuals between 19 and 29 years were granted temporary disability pension in Sweden during 2015, which was more than twice as many individuals in the same age span that received disability pension during 2001 [1]. The pathways to labour market marginalisation are multifaceted, where processes of both health selection and social causation might interact [7–10]. Low attachment to the labour market may lead to development of mental disorders, which in turn may increase the risk of further marginalisation on the labour market, resulting in a viscous circle [11, 12]. There might also be discrepancies between different mental diagnoses regarding granting of disability pension, as reported in a Swedish study [13]. Studies on mental disorders and labour market marginalisation must therefore take previous labour market marginalisation into account [10].

Sweden has since the 1970s, like many other Western countries, experienced increasing immigration. This increase is foremost due to non-Western immigrants from Africa, Asia and South-America, consisting mainly of refugees and relatives seeking reunification, with a more diverse cultural and ethnic origin than the native population [14]. Non-Western immigrants have been shown to have a higher risk for labour market marginalisation compared to the population in the host country [15, 16]. Reasons for this excess risk may comprise differences in sociodemographic and health related factors. Particularly non-Western immigrants seem to a higher extent to have a low educational level as well as jobs with high job strain, but also a higher prevalence of mental disorders [17, 18]. Among individuals with a low educational level, non-Western immigrants have been reported to have an increased risk of being granted disability pension compared to native Swedes [19]. Most of the current literature deals with either the association between immigrant status and mental disorders or immigrant status and labour market marginalisation. There is to date, however, a lack of studies bridging these research fields [15]. Previous findings on social outcomes of a suicide attempt, the most severe consequence of mental disorders, have indicated that pathways to labour market marginalisation might be different for natives and immigrants, i.e. immigrants with a suicide attempt seem to be at a higher risk for unemployment, but at a lower risk for sickness absence and disability pension compared to native Swedes with a suicide attempt [20]. For these reasons, we conceptualised labour market marginalisation from a social insurance perspective, including disability pension, long-term sickness absence and long-term unemployment [21, 22].

The aim was to investigate: 1) the associations between mental disorders, divided into seven subgroups, and three different measures of labour market marginalisation, i.e. disability pension, sickness absence and unemployment and 2) if there were differences regarding labour market marginalisation between immigrants, split in seven subgroups based on region of birth, and native Swedes.

## Methods

### Study population

The study base comprised 1,800,890 non-pensioned young adults between 20 and 35 years of age who were resident in Sweden at 31st of December in 2004. Excluded from the analyses were individuals with granted disability pension before 2004 (*n* = 47,027), and individuals with missing information on region of birth (*n* = 319). A total of 1,753,544 individuals met the inclusion criteria.

### Registers

The study population was identified through registers from Statistics Sweden, consisting of nationwide data for all individuals who were registered as living in Sweden at the 31st of December in 2004. Moreover, register data were obtained for each individual both retrospectively, from 1st of January 2001 and prospectively up to 31st of December 2011 from: 1) Statistics Sweden: age, sex, educational level, family composition, type of living area, date of emigration, labour market attachment and days of unemployment; 2) The Social Insurance Agency: sickness absence and disability pension (date and number of days) from 1994 and onwards; 3) The Swedish National Board of Health and Welfare: date and cause of in- or specialised outpatient health care; date and cause of death.

### Immigrant status

Region of birth was grouped according to the following regions: 1) Sweden; 2) Western countries, mainly consisting of labour immigrants: a) Nordic countries, b) EU except Sweden, Finland and Denmark, c) Europe outside EU excluding former Soviet Union and Nordic countries and d) North America/Oceania, and 3) non-Western countries mainly consisting of refugees and individuals seeking family reunification: a) Africa, b) Asia, including former Soviet Union and c) South-America.

### Variables and diagnostics

#### Exposure measure

The exposure measure was defined as having a mental disorder, measured as a main diagnosis of a mental disorder from in- or specialised outpatient health care between 2001 and 2004. All diagnoses were defined by the corresponding codes of the International Classification of Diseases (ICD), version 10. Mental disorders were classified according to codes F00–99, and somatic disorders were classified according to all remaining codes. When dividing mental diagnoses into subgroups, the chronologically last main diagnosis was used. We divided the diagnoses into: affective disorders (ICD-10 codes F30–39), behavioural, emotional and developmental disorders (ICD-10 codes F50–59, F61-F69, F80-F89, F90-F99), organic disorders/mental retardation (ICD-10 codes F00-F09, F70–79), personality disorders (ICD-10 code F60), schizophrenia/non-affective psychoses (ICD-10 codes F20-F29), neurotic/stress-related/somatoform disorders (ICD-10 codes F40-F48) and substance abuse disorders (ICD-10 codes F10-F19) [20].

#### Outcome measures

The cohort was followed from 2005 to 2011 with regard to the following measures of labour market marginalisation: 1) Disability pension, 2) Long-term sickness absence, defined as ≥90 annual net days of sickness benefit payed by the Swedish Social Insurance Agency during the follow-up period, and 3) Long-term unemployment, defined as ≥180 annual days registered as full time unemployed at the Swedish Public Employment Service during the follow-up period.

#### Covariates

Covariates in the analyses were: I) Socio-demographic factors: sex, age, family composition, type of living area, educational level (all variables were measured at 31st December 2004 and were categorised as indicated in Table 1); II) Measurements of health and labour market marginalisation: In analyses regarding all outcome measures: comorbid somatic disorders, defined as a record of a somatic disorder as main diagnosis from in- or specialised outpatient health care 2004–2011 and labour market attachment, measured during 2004 and categorised as a) income from work, b) income, but not from work and c) no income, were included. In analyses regarding disability pension as outcome measure also annual long-term unemployment 2004–2010 was considered as a covariate; In analyses regarding long-term sickness absence as outcome measure, also long-term sickness absence 2004 and annual long-term unemployment 2004–2010 were used as covariates; In analyses regarding long-term unemployment as outcome measure, long-term unemployment 2004 and annual long-term sickness absence 2004–2010 were included as covariates additionally.

**Table 1:** Characteristics^a^ of individuals^b^ stratified according to their region of birth^c^

	Sweden	Western countries	Non-Western countries
	N (%)	N (%)	N (%)
All	1,480,483 (84.4)	131,486 (7.5)	141,575 (8.1)
Characteristics			
Socio-demographic factors			
Sex			
Men	764,667 (51.7)	64,809 (49.3)	65,083 (46.0)
Women	715,816 (48.3)	66,677 (50.7)	76,492 (54.0)
Age			
20–24 years	439,880 (29.7)	30,737 (23.4)	40,465 (28.6)
25–29 years	446,275 (30.1)	41,688 (31.7)	45,010 (31.8)
30–35 years	594,328 (40.1)	59,061 (44.9)	56,100 (39.6)
Educational level			
High	559,590 (37.8)	47,983 (36.5)	41,995 (29.7)
Medium	789,499 (53.3)	49,103 (37.3)	54,941 (38.8)
Low	128,350 (8.7)	18,177 (13.8)	30,318 (21.4)
Unknown	3044 (0.2)	16,223 (12.3)	14,321 (10.1)
Family composition^d^			
Married/cohabiting with no children	45,751 (3.1)	11,487 (8.7)	12,983 (9.2)
Married/ cohabiting with children	410,268 (27.7)	44,700 (34.0)	43,987 (31.1)
Single with no children	970,912 (65.6)	68,687 (52.2)	75,709 (53.5)
Single with children	53,550 (3.6)	6611 (5.0)	8889 (6.3)
Unknown	2	1	7
Type of living area ^e^			
Big cities	571,240 (38.6)	69,602 (52.9)	82,942 (58.6)
Medium sized cities	542,218 (36.6)	40,849 (31.1)	42,911 (30.3)
Small town	367,025 (24.8)	21,035 (16.0)	15,722 (11.1)
Measurements of health and labour market marginalisation			
Unemployment			
No unemployment	1,170,358 (79.1)	100,368 (76.3)	99,681 (70.4)
1–179 days	260,378 (17.6)	24,634 (18.7)	33,003 (23.3)
≥ 180 days	49,747 (3.4)	6484 (4.9)	8891 (6.3)
Sickness absence			
No sickness absence	1,334,578 (90.2)	120,252 (91.5)	130,594 (92.3)
1–89 days	101,852 (6.9)	7614 (5.8)	7355 (5.2)
≥ 90 days	43,725 (3.0)	3601 (2.7)	3608 (2.5)
Mental disorders	50,795 (3.4)	4388 (3.3)	5748 (4.1)
Affective disorders	12,742 (0.9)	987 (0.8)	1270 (0.9)
Behavioural/emotional/developmental disorders	5323 (0.4)	362 (0.3)	525 (0.4)
Organic disorders/mental retardation	353 (0.0)	31 (0.0)	52 (0.0)
Personality disorders	1345 (0.1)	95 (0.1)	109 (0.1)
Schizophrenia/non-affective disorders	1387 (0.1)	209 (0.2)	337 (0.2)
Neurotic/stress-related/somatoform disorders	17,447 (1.2)	1844 (1.4)	2274 (1.6)
Substance abuse disorders	12,198 (0.8)	860 (0.7)	1181 (0.8)
Somatic disorders	917,036 (61.9)	70,588 (53.7)	88,371 (62.4)

^a^All covariates were measured at baseline (2004) with exception of inpatient and specialised outpatient health care, which was measured 2001–2004

^b^All individuals were 20–35 years, and resident in Sweden at 31st of December 2004 (*N* = 1,753,544)

^c^Western countries (a. Nordic countries except Sweden, b. EU except Sweden, Finland and Denmark, c. Europe outside EU and Nordic countries and d. North America and Oceania); non-Western countries (a. Africa, b. Asia and c. South-America)

^d^With or without children living at home

^e^Type of living area: big cities = Stockholm, Gothenburg, and Malmö; medium-sized cities = cities with >90,000 inhabitants within 30 km distance from the centre of the city; small cities/villages = all remaining cities/villages

### Social Insurance in Sweden

In Sweden, all individuals between 30 and 64 years of age, who due to disease or injury have a permanently impaired work capacity, can be granted disability pension. Individuals between 19 and 29 years can be granted time-restricted disability pension if work capacity is reduced, or if compulsory education is not completed before 19 years of age. All individuals from the age of 16, and with an income from work or benefits from e.g. unemployment or parental insurances, who due to disease or injury have a reduced work capacity, can receive sickness absence benefits. All individuals over 20 years can receive basic levels of unemployment benefit while registered as a job seeker at the Swedish Public Employment Service. Income-related unemployment benefit presupposes, however, earlier income from work.

### Statistics

Cox regression models with both fixed and time-dependent covariates and competing risks were applied. Hazard ratios (HRs) with 95% confidence intervals (CIs) were computed for individuals with mental disorders between 2001 and 2004, both dichotomised and categorised into seven subgroups as stated above, in comparison to individuals without mental disorders during the specified time period. Furthermore, the analyses compared groups of individuals originating from three major regions, divided into 7 sub-regions, with regard to a record of previous mental disorder. Death and emigration were modelled as competing events in all regression models. In the analyses with regard to long-term sickness absence and long-term unemployment as outcome measures, also disability pension was modelled as a competing event. Information on somatic disorders during baseline and the follow-up period was introduced as a dichotomised time-dependent covariate in analyses regarding all outcome measures. Parts of the analyses were also stratified by labour market attachment during 2004. In the analyses with regard to long-term sickness absence and disability pension as outcome measures, information on long-term unemployment during baseline and during the follow-up period was additionally introduced as a time dependent covariate. In the analyses with regard to long-term unemployment as the outcome measure, long-term sickness absence was modelled as a time dependent covariate in addition. Furthermore, tests for effect modification between mental disorders and immigrant status, regarding all three outcome measures, were applied through the partial likelihood ratio test [23]. All analyses were conducted by SAS Statistical Software, version 9.4.

## Results

The cohort included 1,480,483 native Swedes (84.4%), 131,486 Western immigrants (7.5%) and 141,575 non-Western immigrants (8.1%). Among native Swedes, there was a higher proportion of men compared to women, while the opposite was found among both Western and non-Western immigrants (Table 1). Immigrants from Western countries were in general older, compared to both native Swedes and immigrants from non-Western countries. Immigrants, both from Western and non-Western countries, had a lower educational level compared to native Swedes. A higher proportion of immigrants was living in big cities compared to native Swedes and a higher proportion of native Swedes was single without children living at home compared to both immigrant groups. Long-term unemployment at baseline was more common among immigrants, mainly among immigrants from non-Western countries, than among native Swedes. On the contrary, long-term sickness absence at baseline was more common among native Swedes than among immigrants. Mental disorders between 2001 and 2004 were more common among immigrants from non-Western countries (4.1%) compared to both immigrants from Western countries (3.3%) and native Swedes (3.4%). Particularly neurotic/stress-related/somatoform disorders showed this pattern. Also somatic disorders were more common among non-Western immigrants compared to Western immigrants.

The univariate analyses showed a diverging pattern for the associations between mental disorders and the three different measures of labour market marginalisation; disability pension (HR: 11.6), long-term sickness absence (HR: 3.6) and long-term unemployment (HR: 2.2) (Table 2). In the multivariate analyses, sociodemographic factors and measures of health and labour market marginalisation, foremost previous labour market attachment, nearly halved the HRs for all outcome measures; disability pension (HR: 6.7), long-term sickness absence (HR: 2.0) and long-term unemployment (HR: 1.2). When categorising mental disorders into different subgroups, a divergent pattern emerged. Individuals with personality disorders or schizophrenia/non-affective psychoses had higher risk estimates for both disability pension and sickness absence compared to individuals without mental disorders. There were, however, similar increased risks of unemployment among all seven subgroups of mental disorders, compared to individuals without mental disorders. Sociodemographic factors and measurements of health and labour market marginalisation explained almost half of the increased risk for most subgroups of mental disorders, and nearly two thirds of the association between personality disorder and disability pension. The partial likelihood ratio test revealed an effect modification between region of birth and mental disorder regarding disability pension (*p* = 0.004), sickness absence (*p* < 0.001) and unemployment (*p* < 0.001).

**Table 2:** Hazard ratios (HRs)^a^ with 95% confidence intervals (CIs) for disability pension, sickness absence and unemployment for individuals with or without mental disorders 2001–2004^b, c^

	Univariate analysis	Multivariate analysis	
		HR (95%CI)	
Disability pension		Model 1^d^	Model 2^e^
No mental disorders	1	1	1
Mental disorders	11.63 (11.34–11.93)	9.01 (8.78–9.25)	6.73 (6.55–6.91)
Affective disorders	13.38 (12.82–13.96)	10.42 (9.98–10.88)	8.18 (7.83–8.54)
Behavioural/emotional/developmental disorders	9.47 (8.78–10.21)	8.54 (7.92–9.21)	7.16 (6.64–7.72)
Organic disorders/mental retardation	23.75 (19.71–28.62)	19.37 (16.07–23.34)	14.37 (11.92–17.32)
Personality disorders	29.71 (27.13–32.53)	20.08 (18.32–22.00)	12.55 (11.45–13.75)
Schizophrenia/non-affective psychoses	45.63 (42.58–48.89)	38.85 (36.25–41.64)	25.33 (23.62–27.17)
Neurotic/stress-related/somatoform disorders	10.24 (9.84–10.67)	7.71 (7.40–8.03)	6.03 (5.79–6.29)
Substance abuse disorders	7.24 (6.84–7.66)	5.38 (5.08–5.70)	3.52 (3.32–3.73)
Long-term sickness absence			
No mental disorders	1	1	1
Mental disorders	3.61 (3.53–3.68)	3.11 (3.05–3.18)	2.04 (2.00–2.09)
Affective disorders	4.53 (4.37–4.69)	3.73 (3.60–3.86)	2.09 (2.01–2.16)
Behavioural/emotional/developmental disorders	2.87 (2.68–3.06)	2.62 (2.45–2.80)	1.91 (1.78–2.04)
Organic disorders/mental retardation	3.63 (2.89–4.57)	3.52 (2.80–4.43)	2.11 (1.68–2.65)
Personality disorders	5.36 (4.83–5.94)	4.22 (3.81–4.69)	2.60 (2.34–2.89)
Schizophrenia/non-affective psychoses	5.47 (4.98–6.01)	5.56 (5.06–6.11)	3.16 (2.87–3.47)
Neurotic/stress-related/somatoform disorders	3.64 (3.53–3.76)	2.92 (2.83–3.02)	1.91 (1.85–1.98)
Substance abuse disorders	2.62 (2.50–2.74)	2.54 (2.43–2.66)	2.07 (1.98–2.17)
Long-term unemployment			
No mental disorders	1	1	1
Mental disorders	2.18 (2.14–2.21)	1.72 (1.69–1.75)	1.23 (1.21–1.25)
Affective disorders	2.11 (2.04–2.18)	1.78 (1.72–1.84)	1.29 (1.25–1.33)
Behavioural/emotional/developmental disorders	1.70 (1.60–1.80)	1.55 (1.47–1.64)	1.27 (1.20–1.34)
Organic disorders/mental retardation	1.99 (1.62–2.44)	1.56 (1.27–1.92)	1.19 (0.97–1.47)
Personality disorders	2.50 (2.26–2.77)	1.77 (1.60–1.96)	1.17 (1.06–1.30)
Schizophrenia/non-affective psychoses	2.60 (2.37–2.85)	2.15 (1.96–2.36)	1.29 (1.17–1.41)
Neurotic/stress-related/somatoform disorders	2.08 (2.03–2.14)	1.70 (1.65–1.75)	1.27 (1.23–1.30)
Substance abuse disorders	2.53 (2.45–2.61)	1.69 (1.64–1.75)	1.12 (1.09–1.16)

^a^All analyses were conducted by Cox regression with both fixed and time dependent covariates and competing risks

^b^All individuals were 20–35 years old, and resident in Sweden at 31st of December 2004 (*N* = 1,753,544)

^c^Western countries (a. Nordic countries except Sweden, b. EU except Sweden, Finland and Denmark, c. Europe outside EU and Nordic countries and d. North America and Oceania); non-Western countries (a. Africa, b. Asia and c. South-America)

^d^Model 1: Age, sex, educational level, family composition, type of living area

^e^Model 2: Outcome disability pension: Model 1 + somatic disorder 2004–2010, labour market attachment in 2004, ≥180 days of unemployment annually 2004–2010. Outcome long-term sickness absence: Model 1 + somatic disorder 2004–2010, labour market attachment in 2004, ≥180 days of unemployment annually 2004–2010, sickness absence 2004 (0, 1–89, ≥90 days) Outcome long-term unemployment: Model 1 + somatic disorder 2004–2010, labour market attachment in 2004, ≥90 days of sickness absence annually 2004–2010, unemployment 2004 (0, 1–179, ≥180 days)

When stratifying the analyses by labour market attachment during 2004, there was a gradient showing that immigrants, both from Western and non-Western countries, had lower risk estimates for all outcome measures the more distant they were from having income from work (Table 3). Among persons with income from work, immigrants had higher risk estimates for disability pension and long-term sickness absence while persons with income only from benefits or no income at all had lower risk estimates regarding those outcome measures compared to native Swedes. This gradient was not that accentuated regarding long-term unemployment.

**Table 3:** Multivariate hazard ratios (HRs)^a^ with 95% confidence intervals (CIs) for disability pension, sickness absence and unemployment and region of birth^b,c^ stratified on labour market attachment

	Region of birth	Disability pension	Long-term sickness absence	Long-term unemployment
			HR (95% CI)	
Income from work during 2004				
No mental disorders	Sweden	1	1	1
	Western countries	1.55 (1.45–1.67)	1.29 (1.25–1.33)	2.04 (2.00–2.09)
	Non-Western countries	1.36 (1.26–1.47)	1.35 (1.31–1.39)	2.45 (2.40–2.50)
Mental disorders	Sweden	8.96 (8.54–9.39)	2.20 (2.13–2.26)	1.67 (1.62–1.72)
	Western countries	10.35 (8.95–11.97)	2.23 (2.02–2.47)	2.78 (2.53–3.05)
	Non-Western countries	9.09 (7.82–10.56)	2.15 (1.94–2.37)	2.62 (2.40–2.85)
Income, but not from work, during 2004				
No mental disorders	Sweden	1	1	1
	Western countries	0.70 (0.60–0.81)	0.98 (0.92–1.05)	1.36 (1.32–1.40)
	Non-Western countries	0.72 (0.63–0.81)	0.92 (0.87–0.97)	1.66 (1.62–1.70)
Mental disorders	Sweden	6.68 (6.22–7.18)	2.43 (2.30–2.56)	1.28 (1.23–1.33)
	Western countries	4.57 (3.60–5.79)	2.06 (1.72–2.46)	1.57 (1.40–1.75)
	Non-Western countries	5.74 (4.80–6.86)	2.28 (1.98–2.63)	1.72 (1.57–1.88)
No income during 2004				
No mental disorders	Sweden	1	1	1
	Western countries	0.42 (0.39–0.46)	0.71 (0.66–0.75)	1.15 (1.12–1.18)
	Non-Western countries	0.39 (0.36–0.42)	0.60 (0.56–0.63)	1.59 (1.56–1.62)
Mental disorders	Sweden	4.54 (4.35–4.74)	1.55 (1.47–1.64)	1.13 (1.11–1.17)
	Western countries	3.02 (2.71–3.38)	1.21 (1.04–1.41)	1.33 (1.23–1.44)
	Non-Western countries	3.15 (2.87–3.46)	1.11 (0.96–1.28)	1.72 (1.61–1.84)

^a^All analyses were conducted by Cox regression with both fixed and time dependent covariates and competing risks. Multivariate model: Outcome disability pension; Model 1 + somatic disorder 2004–2010, ≥180 days of unemployment annually 2004–2010. Outcome long-term sickness absence; Model 1 + somatic disorder 2004–2010, ≥180 days of unemployment annually 2004–2010, sickness absence 2004 (0, 1–89, ≥90 days) Outcome long-term unemployment; Model 1 + somatic disorder 2004–2010, ≥90 days of sickness absence annually 2004–2010, unemployment 2004 (0, 1–179, ≥180 days).

^b^All individuals were 20–35 years, and resident in Sweden at 31st of December 2004 (*N* = 1,753,544)

^c^Western countries (a. Nordic countries except Sweden, b. EU, except Sweden, Finland and Denmark, c. Europe outside EU and Nordic countries and d. North America and Oceania); non-Western countries (a. Africa, b. Asia and c. South-America)

When categorising individuals with respect to region of birth, the univariate analyses revealed that immigrants without mental disorders had a slightly higher risk for disability pension compared to native Swedes (Table 4). In the multivariate analyses, foremost when previous labour market attachment was taking into account, the risk of disability pension among both Western and non-Western immigrants was lower compared to native Swedes. Lowest risk estimates for disability pension were found among individuals from EU, North America/Oceania and Africa. Among persons without mental disorders, the HRs regarding long-term sickness absence were slightly higher among immigrants than among native Swedes, both in the univariate and in the full model (model 2). In the full model, immigrants from Europe outside EU had the highest risk estimates of long-term sickness absence while immigrants from Africa and North America/Oceania had the lowest risk estimates compared to native Swedes. Regarding unemployment, results of the univariate analyses revealed a gradient: immigrants from non-Western countries had highest risk estimates for subsequent long-term unemployment, followed by immigrants from Western countries and finally native Swedes. The multivariate analyses confirmed this pattern, but on a lower level of effect sizes. Nordic immigrants had lower risk estimates of long-term unemployment while all other immigrant groups had higher risk estimates of long-term unemployment compared to native Swedes, highest estimates were seen among immigrants from Europe outside EU, Asia and Africa.

**Table 4:** Hazard ratios (HRs)^a^ with 95% confidence intervals (CIs) for disability pension, sickness absence and unemployment and region of birth^b,c^

	Region of birth	Univariate analysis	Multivariate analyses	
			Model 1^d^	Model 2^e^
		HR (95% CI)		
Disability pension				
No mental disorders	Sweden	1	1	1
	Western countries	1.25 (1.19–1.31)	1.15 (1.09–1.21)	0.81 (0.77–0.85)
	Nordic countries	1.17 (1.05–1.31)	1.04 (0.93–1.15)	0.80 (0.72–0.90)
	EU	0.82 (0.74–0.92)	0.86 (0.77–0.96)	0.60 (0.53–0.66)
	Europe outside EU	1.61 (1.51–1.71)	1.37 (1.29–1.46)	0.95 (0.89–1.01)
	North America/Oceania	0.70 (0.55–0.88)	0.75 (0.59–0.95)	0.49 (0.38–0.61)
	Non-Western countries	1.28 (1.22–1.34)	1.07 (1.02–1.12)	0.65 (0.62–0.68)
	South America	1.07 (0.94–1.21)	0.99 (0.87–1.13)	0.76 (0.67–0.86)
	Asia	1.37 (1.30–1.44)	1.14 (1.08–1.21)	0.68 (0.65–0.72)
	Africa	1.07 (0.96–1.21)	0.81 (0.72–0.91)	0.47 (0.42–0.53)
Mental disorders	Sweden	12.08 (11.75–12.42)	9.32 (9.05–9.59)	6.55 (6.36–6.75)
	Western countries	12.24 (11.29–13.28)	8.42 (7.76–9.14)	4.80 (4.42–5.22)
	Nordic countries	14.04 (11.84–16.66)	9.11 (7.68–10.81)	5.47 (4.61–6.49)
	EU	11.53 (9.70–13.71)	8.92 (7.50–10.61)	5.25 (4.41–6.25)
	Europe outside EU	12.26 (10.96–13.71)	8.00 (7.14–8.97)	4.40 (3.93–4.94)
	North America/Oceania	9.53 (6.62–13.71)	7.91 (5.50–11.39)	4.77 (3.31–6.86)
	Non-Western countries	11.97 (11.14–12.87)	8.57 (7.97–9.22)	4.76 (4.42–5.13)
	South America	10.86 (9.01–13.1)	8.13 (6.73–9.80)	5.33 (4.42–6.43)
	Asia	11.81 (10.84–12.87)	8.58 (7.87–9.35)	4.64 (4.25–5.06)
	Africa	13.93 (11.68–16.61)	8.90 (7.46–10.62)	4.80 (4.02–5.73)
Long-term sickness absence				
No mental disorders	Sweden	1	1	1
	Western countries	1.12 (1.10–1.15)	1.07 (1.05–1.10)	1.15 (1.12–1.18)
	Nordic countries	1.08 (1.02–1.14)	1.05 (1.00–1.11)	1.08 (1.02–1.14)
	EU	0.80 (0.76–0.84)	0.87 (0.82–0.91)	0.96 (0.91–1.01)
	Europe outside EU	1.43 (1.39–1.48)	1.22 (1.18–1.26)	1.30 (1.26–1.34)
	North America/Oceania	0.62 (0.56–0.70)	0.72 (0.64–0.81)	0.82 (0.73–0.92)
	Non-Western countries	1.09 (1.07–1.12)	0.94 (0.92–0.97)	1.07 (1.05–1.10)
	South America	1.10 (1.03–1.17)	1.01 (0.95–1.08)	1.06 (1.00–1.13)
	Asia	1.13 (1.11–1.16)	0.97 (0.95–1.00)	1.11 (1.08–1.14)
	Africa	0.96 (0.91–1.02)	0.79 (0.74–0.83)	0.92 (0.87–0.97)
Mental disorders	Sweden	3.80 (3.72–3.88)	3.28 (3.21–3.36)	2.11 (2.07–2.16)
	Western countries	3.31 (3.07–3.57)	2.52 (2.34–2.72)	1.93 (1.79–2.08)
	Nordic countries	3.71 (3.16–4.36)	2.84 (2.42–3.34)	1.85 (1.57–2.17)
	EU	3.27 (2.80–3.83)	2.73 (2.34–3.20)	2.06 (1.76–2.41)
	Europe outside EU	3.34 (3.01–3.70)	2.39 (2.16–2.66)	1.96 (1.77–2.18)
	North America/Oceania	2.12 (1.47–3.05)	1.89 (1.32–2.73)	1.46 (1.01–2.10)
	Non-Western countries	2.80 (2.61–3.00)	2.20 (2.05–2.36)	1.85 (1.73–1.99)
	South America	2.88 (2.42–3.44)	2.39 (2.00–2.84)	1.85 (1.55–2.20)
	Asia	2.89 (2.66–3.13)	2.27 (2.09–2.46)	1.92 (1.77–2.08)
	Africa	2.27 (1.85–2.79)	1.72 (1.40–2.11)	1.54 (1.25–1.89)
Long-term unemployment				
No mental disorders	Sweden	1	1	1
	Western countries	2.03 (2.01–2.06)	2.09 (2.06–2.12)	1.60 (1.58–1.63)
	Nordic countries	1.03 (0.99–1.07)	1.08 (1.04–1.12)	0.93 (0.89–0.97)
	EU	1.49 (1.45–1.53)	1.66 (1.62–1.71)	1.33 (1.30–1.37)
	Europe outside EU	2.94 (2.89–2.99)	2.82 (2.77–2.87)	2.03 (2.00–2.07)
	North America/Oceania	1.50 (1.41–1.58)	1.68 (1.59–1.78)	1.26 (1.19–1.33)
	Non-Western countries	3.04 (3.01–3.08)	2.87 (2.84–2.91)	2.01 (1.98–2.03)
	South America	2.42 (2.34–2.49)	2.32 (2.25–2.39)	1.75 (1.69–1.80)
	Asia	3.08 (3.04–3.12)	2.94 (2.90–2.98)	2.05 (2.02–2.07)
	Africa	3.44 (3.35–3.52)	3.10 (3.03–3.18)	2.10 (2.05-2.15)
Mental disorders	Sweden	2.41 (2.36–2.46)	1.95 (1.92–1.99)	1.37 (1.34–1.39)
	Western countries	3.65 (3.45–3.85)	2.96 (2.80–3.12)	1.80 (1.71–1.90)
	Nordic countries	2.65 (2.31–3.05)	2.08 (1.81–2.39)	1.39 (1.21–1.59)
	EU	3.24 (2.88–3.64)	2.77 (2.47–3.12)	1.72 (1.53–1.94)
	Europe outside EU	4.27 (3.99–4.59)	3.43 (3.20–3.68)	2.00 (1.87–2.15)
	North America/Oceania	3.44 (2.76–4.28)	3.04 (2.44–3.79)	1.81 (1.45–2.25)
	Non-Western countries	4.19 (4.01–4.38)	3.34 (3.20–3.49)	2.00 (1.91–2.09)
	South America	3.77 (3.35–4.23)	2.92 (2.60–3.28)	1.79 (1.59–2.01)
	Asia	4.24 (4.02–4.46)	3.46 (3.28–3.64)	2.09 (1.98–2.2)
	Africa	4.46 (3.97–5.01)	3.38 (3.01–3.79)	1.91 (1.70–2.15)

^a^All analyses were conducted by Cox regression with both fixed and time dependent covariates and competing risks

^b^All individuals were 20–35 years, and resident in Sweden at 31st of December 2004 (*N* = 1,753,544)

^C^Western countries (a. Nordic countries except Sweden, b. EU, except Sweden, Finland and Denmark, c. Europe outside EU and Nordic countries and d. North America and Oceania); non-Western countries (a. Africa, b. Asia and c. South-America)

^d^Model 1: Age, sex, educational level, family composition, type of living area

^e^Model 2: Outcome disability pension; Model 1 + somatic disorder 2004–2010, labour market attachment in 2004, ≥180 days of unemployment annually 2004–2010. Outcome long-term sickness absence; Model 1 + somatic disorder 2004–2010, labour market attachment in 2004, ≥180 days of unemployment annually 2004–2010, sickness absence 2004 (0, 1–89, ≥90 days) Outcome long-term unemployment; Model 1 + somatic disorder 2004–2010, labour market attachment in 2004, ≥90 days of sickness absence annually 2004–2010, unemployment 2004 (0, 1–179, ≥180 days)

Regarding individuals with mental disorders, risk estimates for disability pension were similar between native Swedes and immigrants in the univariate analyses (Table 4). In the multivariate analyses, foremost when previous labour market attachment was taken into account, both Western and non-Western immigrants had lower risk estimates of disability pension compared to native Swedes. The risk of disability pension was similar among all immigrant subgroups. The univariate analyses showed that native Swedes had a higher risk of long-term sickness absence compared to both Western and non-Western immigrants, but adjustment for sociodemographic factors and measures of health and labour market marginalisation nearly eliminated this difference. The risk estimates of long-term sickness absence given a mental disorder were quite comparable among all immigrant subgroups. The same patterns of associations regarding immigrant status and long-term unemployment reported for individuals without mental disorders were also found for individuals with mental disorders. Adjustment for sociodemographic factors and measures of health and labour market marginalisation decreased the HRs considerably in the multivariate analyses. The risk estimates of long-term unemployment were similar for immigrants from non-Western countries with or without mental disorders and were rather similar among all immigrant subgroups.

## Discussion

Individuals with mental disorders had higher risk estimates for subsequent disability pension (HR; 6.7), long-term sickness absence (HR; 2.0) and long-term unemployment (HR; 1.2) than individuals without mental disorders. Individuals with personality disorders or schizophrenia/non-affective psychoses had highest risk estimates for having disability pension and long-term sickness absence, while the risk estimates of long-term unemployment were similar for all subgroups of mental disorders. Among individuals with mental disorders, the risk estimates for disability pension were lower among immigrants compared to native Swedes. Native Swedes with mental disorders had a slightly higher risk for long-term sickness absence compared to both Western and non-Western immigrants with mental disorders. There was a gradient showing that immigrants from non-Western countries had the highest risk estimates for long-term unemployment followed by immigrants from Western countries and native Swedes, regardless if they had mental disorders or not. The risk estimates were rather similar between sub-regions in both Western and non-Western countries, however with some minor exceptions. The stratified analysis showed that immigrants had higher risk estimates than native Swedes for having disability pension and long-term sickness absence in a cohort of individuals having gainful employment, but lower risk estimates of both long-term sickness absence and disability pension in a cohort of individuals without gainful employment.

The link between mental disorders and having an unstable position on the labour market is well established in earlier research [10, 24]. This study adds to this existing knowledge by measuring three different types of labour market marginalisation and the differences in risk estimates for the association between mental disorders and the various measures were notable. Our findings, along with results from other studies on suicide attempt, confirm that only considering unemployment would underestimate the true consequences of mental disorders in terms of labour market marginalisation [7, 21, 22]. When dividing mental disorders into subgroups, it was evident that individuals with personality disorders and schizophrenia/non-affective psychoses had outstanding high risk estimates for having disability pension, but also high risk estimates for long-term sickness absence compared to individuals with other mental disorders. The hardship in finding and keeping a job on the regular labour market among persons with e.g. schizophrenia and other psychoses is well described in the previous literature [25, 26].

Immigrants from non-Western countries had highest rates of both mental and somatic disorders in 2001–2004. Based on these findings higher risk estimates for subsequent labour market marginalisation based on medical assessments (i.e. sickness absence and disability pension) can be anticipated. Still, our findings suggest the contrary, namely lower risk estimates for both disability pension and long-term sickness absence among immigrants from non-Western countries compared to natives. Treatment before granting of disability pension due to mental disorders has been shown to differ between native Swedes and foremost immigrants from non-Western countries [27]. Moreover, adequate mental health care as well as access to health related benefits like disability pension and sickness absence, which demand certificates from physicians, might also be hampered by language barriers and culturally determined stigmatisation of mental disorders among immigrants [28, 29].

Adjustment for sociodemographic factors reduced the differences in risk estimates between immigrants and native Swedes regarding all outcome measures. Also other studies have reported that sociodemographic factors are of great importance when explaining differences in rates of disability pension between immigrants and the native population [30, 31]. The present study further adds that immigrants had significantly lower risk estimates for disability pension compared to native Swedes when also taking previous labour market attachment into account. This indicates that when an individual has been marginalised on the labour market, there is a high probability that the marginalisation will continue particularly in case of an immigrant background. Marginalisation on the labour market has been shown to be more common among immigrants and joblessness might lead to stress-related mental disorders or other disorders, which in turn might lead to further marginalisation [9, 24, 32]. Immigrants had, compared to native Swedes, higher risk estimates for subsequent unemployment regardless if they had mental disorders or not. Notable was also the small difference between the risk estimates of long-term unemployment among non-Western immigrants with and without mental disorders. One explanation might be the high general risk of unemployment among non-Western immigrants, suggesting considerable difficulties for labour market participation in this group. Mental disorders do not seem to add to this risk. Therefore, processes towards labour market marginalisation seem to differ between native Swedes and immigrants, particularly between native Swedes and immigrants from non-Western countries. One explanation seems to be that immigrants, due to their more unstable situation on the labour market, do not have eligibility for sickness benefits, and limited opportunities to have income-related unemployment benefit and disability pension.

As mentioned above, the construction of the social insurance system might be one of the explanations to discrepancies in labour market marginalisation between native Swedes and immigrant groups. Differences in pathways to different measures of labour market marginalisation may thus be a consequence of the regulations within the social insurance system in Sweden. Moreover, stratification by previous labour market attachment showed that immigrants, in comparison to native Swedes, had decreasing risk estimates for having sickness absence, the more distant they were from having gainful employment. Income from work is a prerequisite for having sickness absence. In Sweden it is, however, possible to be granted basic levels of disability pension and unemployment benefits without earlier income from work. In spite of that, we showed that immigrants had decreasing risk estimates compared to native Swedes also regarding disability pension and unemployment. This may be due to the fact that the basic level of both unemployment benefit and disability pension is on a rather low level, resulting in the fact that some individuals do not seek those benefits after all. Another explanation might be that immigrants do not have adequate knowledge on the structure of the social insurance system, at least during the first years in the new country.

### Strengths and limitations

Strengths of this study include the use of nationwide high quality data from Swedish registers providing large study populations with practically no loss to follow-up [33–35]. Another strength was the inclusion of both health related factors and measures of labour market marginalisation as time-dependent variables in order to consider temporal variation in health and social measures during follow-up. We also considered the fact that the occurrence of one outcome measure (i.e. disability pension) might impede the occurrence of the other outcome measures (i.e. unemployment and sickness absence) by applying regression models considering competing risks. We have also in this study taken previous labour market marginalisation, including degree of attachment to the labour market at baseline, into account, as joblessness may in itself have detrimental effects on later labour market participation [9, 36].

This study had also some limitations. Mental disorders were defined by in- or specialised outpatient care, which mostly reflects medically more serious mental disorders. Individuals treated in primary health care could not be included. In this study immigrants were divided into groups regarding regions of birth country. Those groups are, however, heterogeneous and differences between individuals within these groups are likely to exist. Although the analyses were adjusted for previous labour market marginalisation and stratified for labour market attachment, there might still be remaining residual confounding due to the complicated bidirectional causal pathways between marginalisation on the labour market and mental disorders, reported in previous studies [24, 36].

## Conclusions

Mental disorders among young adults were associated with an increased risk of all three measures of labour market marginalisation, strongest with subsequent disability pension. Native Swedes had higher risk estimates for both disability pension and sickness absence, but lower risk estimates for unemployment compared to both Western and non-Western immigrants, indicating that processes towards labour market marginalisation might differ between native Swedes and immigrants with mental disorders. Previous labour market attachment explained a great part of the association between mental disorders, immigrant status and labour market marginalisation. In studies comparing immigrants with natives with regard to subsequent labour market marginalisation, it is important to consider different measures of subsequent labour market marginalisation in order not to underestimate the consequences of mental disorders. It is, however, also important to consider previous labour market attachment in order not to overestimate the differences between immigrants and native Swedes.
